# Two decades of molecular surveillance in Senegal reveal rapid changes in known drug resistance mutations over time

**DOI:** 10.1186/s12936-024-05024-8

**Published:** 2024-07-09

**Authors:** Yaye D. Ndiaye, Wesley Wong, Julie Thwing, Stephen F. Schaffner, Katelyn Vendrely Brenneman, Abdoulaye Tine, Mamadou A. Diallo, Awa B. Deme, Mouhamad Sy, Amy K. Bei, Alphonse B. Thiaw, Rachel Daniels, Tolla Ndiaye, Amy Gaye, Ibrahima M. Ndiaye, Mariama Toure, Nogaye Gadiaga, Aita Sene, Djiby Sow, Mamane N. Garba, Mamadou S. Yade, Baba Dieye, Khadim Diongue, Daba Zoumarou, Aliou Ndiaye, Jules F. Gomis, Fatou B. Fall, Medoune Ndiop, Ibrahima Diallo, Doudou Sene, Bronwyn Macinnis, Mame C. Seck, Mouhamadou Ndiaye, Bassirou Ngom, Younouss Diedhiou, Amadou M. Mbaye, Lamine Ndiaye, Ngayo Sy, Aida S. Badiane, Daniel L. Hartl, Dyann F. Wirth, Sarah K. Volkman, Daouda Ndiaye

**Affiliations:** 1 International Research Training Center On Genomics and Health Surveillance (CIGASS), Cheikh Anta Diop University, 16477 Dakar, Senegal; 2 Department of Immunology and Infectious Diseases, Harvard T.H. Chan School of Public Health, 665 Huntington Ave, Boston, MA 02115 USA; 3 Malaria Branch, Division of Parasitic Diseases and Malaria, Global Health Center, Centers for Disease Control and Prevention, Atlanta, GA USA; 4 Broad Institute of MIT and Harvard, 415 Main Street, Cambridge, MA 02142 USA; 5 Yale School of Public Health, 60 College St, New Haven, CT 06510 USA; 6 Department of Biochemistry and Functional Genomics, Sherbrooke University, 2500 Bd de L’Universite, Sherbrooke, QC J1K 2R1 Canada; 7 RNA Therapeutics Institute, UMass Chan Medical School, 368 Plantation Street, Worcester, MA 01605 USA; 8 National Malaria Control Programme (NMCP), 25270 Dakar, Senegal; 9 Service de Lutte Antiparasitaire (SLAP), Thiès, Senegal; 10 Department of Organismic and Evolutionary Biology, Harvard University, 16 Divinity Avenue, Cambridge, MA 02138 USA; 11 Simmons University, 300 The Fenway, Boston, MA 02115 USA

## Abstract

**Background:**

Drug resistance in *Plasmodium falciparum* is a major threat to malaria control efforts. Pathogen genomic surveillance could be invaluable for monitoring current and emerging parasite drug resistance.

**Methods:**

Data from two decades (2000–2020) of continuous molecular surveillance of *P. falciparum* parasites from Senegal were retrospectively examined to assess historical changes in malaria drug resistance mutations. Several known drug resistance markers and their surrounding haplotypes were profiled using a combination of single nucleotide polymorphism (SNP) molecular surveillance and whole genome sequence based population genomics.

**Results:**

This dataset was used to track temporal changes in drug resistance markers whose timing correspond to historically significant events such as the withdrawal of chloroquine (CQ) and the introduction of sulfadoxine-pyrimethamine (SP) in 2003. Changes in the mutation frequency at *Pfcrt* K76T and *Pfdhps* A437G coinciding with the 2014 introduction of seasonal malaria chemoprevention (SMC) in Senegal were observed. In 2014, the frequency of *Pfcrt* K76T increased while the frequency of *Pfdhps* A437G declined. Haplotype-based analyses of *Pfcrt* K76T showed that this rapid increase was due to a recent selective sweep that started after 2014.

**Discussion (Conclusion):**

The rapid increase in *Pfcrt* K76T is troubling and could be a sign of emerging amodiaquine (AQ) resistance in Senegal. Emerging AQ resistance may threaten the future clinical efficacy of artesunate-amodiaquine (ASAQ) and AQ-dependent SMC chemoprevention. These results highlight the potential of molecular surveillance for detecting rapid changes in parasite populations and stress the need to monitor the effectiveness of AQ as a partner drug for artemisinin-based combination therapy (ACT) and for chemoprevention.

**Supplementary Information:**

The online version contains supplementary material available at 10.1186/s12936-024-05024-8.

## Background

The World Health Organization (WHO) estimated 247 million malaria cases and 619,000 malaria deaths in 2021 [1]. Children under five years of age are the most vulnerable to malaria, accounting for 80% of deaths worldwide, with 96% of all malaria cases and deaths in 2021 occurring in the African region [1]. Increased funding for malaria control has led to an estimated 30% reduction of malaria mortality since 2000, using a combination of vector control and drug-based interventions [1]. However, the development of parasite resistance to anti-malarials threatens to undermine control and elimination efforts and poses a significant threat to public health. Anti-malarials are used for both therapeutic treatment and chemoprevention. Chemoprevention includes intermittent preventive treatment for pregnant women (IPTp) and infants (IPTi), seasonal malaria chemoprevention (SMC), and mass drug administration (MDA) [2].

Pathogen molecular surveillance tracks emerging trends in parasite drug resistance, which the WHO defines as the ability of a parasite to survive or multiply despite the administration and absorption of a drug given in doses equal to or higher than the recommended therapeutic dose [3]. Therapeutic efficacy studies (TES) are the gold standard for evaluating clinical and therapeutic drug efficacy [3, 4] but are resource intensive and challenging to implement. Pathogen molecular surveillance of known drug resistance markers can be used to assess the risk of emerging drug resistance or changes in parasite fitness [5–10] before clinical drug efficacy is irreparably compromised. Of particular importance is the continuous evaluation of drug resistance mutations for artemisinin (ART) and partner drugs used in artemisinin-based combination therapy (ACT). ART (partial) resistance is associated with delayed clearance times in Southeast Asia and is defined as having more than 10% of patients with asexual parasites on the third day following treatment [3]. Molecular surveys of drug resistance include the monitoring of single nucleotide polymorphisms (SNPs), copy number variations associated with drug resistance [1, 10], and haplotype-based analyses of hard or soft selective sweeps [10, 11]. Drug resistance markers are often associated with selective sweeps [12] as they provide a strong survival advantage to parasites that harbor the drug resistance alleles under drug pressure.

Previous studies have identified multiple molecular markers associated with antimalarial drug resistance. Chloroquine (CQ) resistance has been associated with changes in the chloroquine resistance transporter gene *Pfcrt*, particularly at codon *Pfcrt *K76T [13, 14]. Sulfadoxine-pyrimethamine (SP) resistance is associated with mutations at codons 51, 59 and 108 of dihydrofolate reductase (*Pfdhfr*) and codons 437 and 540 of dihydropteroate synthase (*Pfdhps*) [15, 16]. Mutations in the multi-drug resistance *Pfmdr1* gene that have been associated with resistance against many drugs, including amodiaquine (AQ), mefloquine, quinine, lumefantrine and other quinolines [17–19]. Mutations in the kelch propeller domain gene *Pfkelch13* have been associated with ART resistance and delayed parasite clearance [3, 20].

This study examined a two-decades long collection of *P. falciparum* samples obtained from febrile individuals in Senegal to identify historical changes in drug resistance markers. Based on the historical anti-malarial policy in Senegal, this study focused surveying WHO-validated and verified [4] SNPs in *Pfcrt, Pfdhfr, Pfdhps, and Pfmdr1* associated with resistance to CQ, AQ, and SP [3]. Molecular surveillance of *Pfkelch13* was started in 2015 in response to the emergence of *Pfkelch13* C580Y as a marker associated with partial ART resistance markers in Southeast Asia [20–22] and to monitor the frequency of *Pfkelch13* A578S, which is no longer considered to be associated with resistance but at the time was the most frequent mutation reported in Africa [23]. The goal of this study was to determine whether there were changes in parasite population genetics that could serve as an early warning sign for emerging drug resistance or changes in parasite fitness.

## Methods

### Sampling

Parasite samples were collected from treatment seeking patients aged 3 months or older presenting with fever or history of fever within the prior 48 h at clinics in Pikine, Thiès, Kédougou, Diourbel, Kolda, and Kaolack between 2000 and 2020. Informed consent was obtained from all patients, or from their parents or guardians if the patient was a minor. All patients with positive tests for malaria received free malaria treatment with AL or ASAQ, in accordance with the National Health Development Policy in Senegal as recommended by the WHO.

Microscopy slides, rapid diagnostic tests, and finger stick blood samples, (dried blood sample, DBS) spotted on (Whatman Protein Saver FTA (Whatman^®^ 3MM CHR CAT N° 3030–662) filter paper, were obtained from all consenting individuals. Samples were stored in plastic bags at room temperature and protected with silica gel desiccant before DNA isolation and molecular testing. All samples were coded and anonymous, as per the consent process.

### Laboratory procedures

#### Sample collection and DNA extraction

For the SNP-based molecular surveillance data, a total of 3284 DNA samples were extracted from DBS using QIAamp DNA Mini Kit (QIAGEN, Valencia, CA, USA), according to manufacturer’s directions. These samples were genotyped using high-resolution melting (HRM) or targeted amplicon genome (TAG) sequencing (see below).

### High-resolution melting

Drug resistance markers in *Pfcrt*, *Pfmdr1*, *Pfdhfr*, *Pfdhps* were assessed using a high resolution melting (HRM) assay with the Roche LightCycler 96 instrument (Roche Molecular systems) as previously described [24]. The HRM assay was set up in a total volume of 5 μl containing 2.5 μl of DNA and 2.5 × LightScanner mastermix LCGreen (Plus double-stranded DNA dye (Idaho Technology, Inc.)). The codons 76 in *Pfcrt* gene; 86, 184, 1042, 1246 in *Pfmdr1* gene; 51, 59, 108 in *Pfdhfr* gene; 437, 540, 581, 613 in *Pfdhps* gene were used. The following reference DNA strains were used: 3D7, Dd2, HB3, and IPC_3445 (MRA-1236).

### Amplification and sequencing of the Pfkelch13 gene

The *Pfkelch13* propeller domain was amplified using a *P. falciparum* specific protocol described previously [25]. PCR products were visualized on a 2% agarose gel after electrophoresis. Sequencing of PCR products was performed using an ABI 3730 sequencer by Sanger using a protocol established at CDC/Atlanta Malaria Genomic laboratory (Applied Biosystems, Foster City, CA).

### Data analysis

The HRM result was analyzed using the LightCycler 96 application software version 1.1.0.1320. The *Pfkelch13* sequence data was analyzed using the Geneious software (version 9.0.5, www.geneious.com). A cutoff of quality score HQ (High Quality) > 30% was applied to all sequences. Polymorphisms were considered if both the forward and reverse strands carried a mutation and matched the quality score cut off.

### Haplotype analyses

A subset of 231 monogenomic (single-strain) samples collected from febrile, clinic-reporting patients between 2006 and 2019 from Pikine, Thiès, and Kédougou were previously whole genome sequenced using next-generation Illumina short reads [26–28]. These sequences excluded polygenomic (multiple strain) infections and avoided repeated sequencing of clones using a 24-SNP molecular barcode [29]. Short-reads were aligned to the *P. falciparum* 3D7 reference genome (PlasmoDB v. 28) using BWA-mem and Picard Tools. An initial set of 577487 genome-wide SNPs were called using HaplotypeCaller in GATK v3.5 using the quality filters and standards for best practices as described by the Pf3k project [30, 31]. Bifurcation plots and extended haplotypes were calculated using *rehh 2.0* [32].

The genomic regions surrounding *Pfcrt, Pfdhfr, and Pfdhps* were defined as the genomic region 100 kb upstream and downstream of the starting and ending boundaries (obtained from PlasmoDB, Plasmodb.org) of each gene. For each genomic region, samples and variant sites were filtered by sequentially applying the following filters: (1) first removing samples with > 50% unusable data (missing, heterozygous, or triallelic sites), (2) then retaining sites with < 10% unusable data, and (3) finally removing samples with > 5% unusable data. These filters were applied separately across all sample years, for samples collected before or during 2014 (pre-SMC), and for samples collected after 2014 (post-SMC).

### Generalized additive model

Binomial generalized additive models were fit using the R package *mcgv* (v1.8–40). The structure of the GAM was defined as:

$$f\left( {proportion_{mutant} } \right) = s\left( {year, k} \right) + is_{Diourbel} + is_{Kedougou } + is_{Dakar} + is_{Thies}$$

where $proportion_{mutant}$ is the proportion of samples with the mutant allele, *s*(*year,k*) is a smoothing spline function for the sample year. The *k* is the degrees of freedom used in the smoothing spline function. The other covariates are binary categorical variables specifying sample origin. Kolda and Kaolack were excluded because they were sampled for less than three years.

### H12 and H2/H1 analyses

A total of 43386 SNPs were used for H12 and H2/H1 analyses. These SNPs were optimized to accurately estimate genetic relatedness based on identity-by-descent (IBD) estimation and are described in detail in a previous publication [28]. Briefly, SNPs were identified after dividing the genome into 2 kb windows and choosing SNPs such that each window contained a maximum of 12 SNPs, requiring SNPs have a minimum allele frequency of 0.5%, requiring that markers be at least 20 bp apart [28]. These SNPs were used to identify haplotypes based on the shared chromosome segments that were IBD using the program hmmIBD (version 2.0.4) [33] after setting the genome-wide IBD fraction parameter to 20% to better assess local IBD status in parasites that otherwise have high genome-wide IBD sharing.

At each SNP locus, haplotypes were defined by clustering samples that were related to one another. Clustering was performed via a greedy algorithm as follows. For a pair of samples IBD at a SNP site:

- If neither is already in a cluster, form a new cluster.
- If both are already in the same cluster, do nothing.
- If they are already in different clusters, merge the clusters if 40% of pairwise comparisons are IBD.
- If one is in a cluster, add the other if it is IBD with at least 40% of existing samples in the cluster. Otherwise, start a new cluster.

The resulting clusters are defined as the set of haplotypes at that locus. H1, H2, and H12 [34, 35] were calculated as:

$$H_{1} = p_{1}^{2} + p_{2}^{2} + \ldots$$

$$H_{2} = p_{2}^{2} + p_{3}^{2} + \ldots$$

$$H_{12} = 2p_{1} p_{2} + p_{1}^{2} + p_{2}^{2} + p_{3}^{2} + \ldots$$

where p_i_ is the frequency of the i-th most frequent haplotype. H12 treats the two most common haplotypes as a single haplotype. Singleton haplotypes were omitted since their population frequency is likely to be much lower than their sample frequency.

## Results

### Study design and SNP-based molecular surveillance sampling

**Fig. 1:**
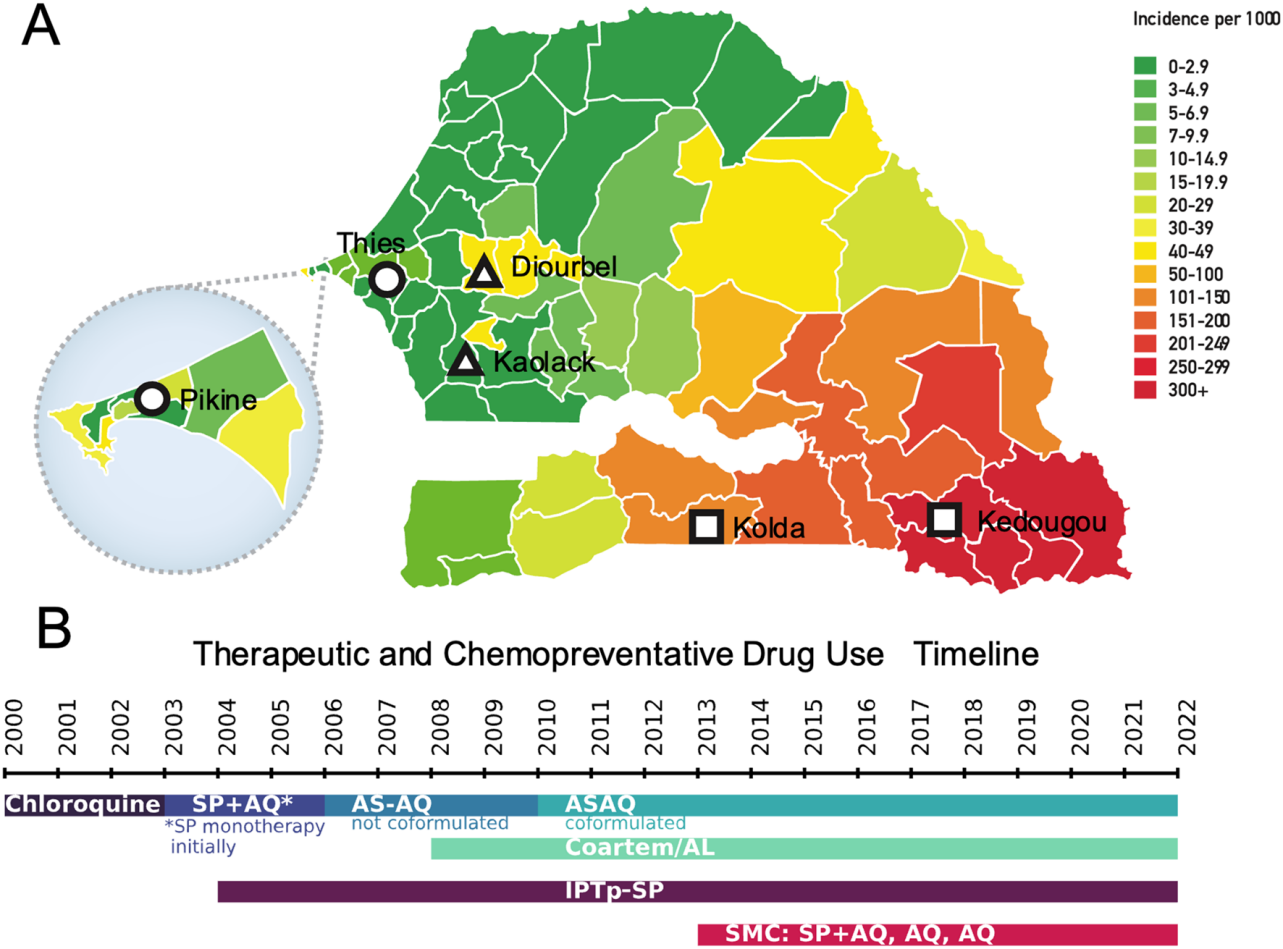
Study design and Senegal drug history **A** Map of Senegal highlighting the different study locations and their corresponding transmission levels in 2019. Each region is colored by transmission intensity, with red indicating high transmission and green indicating low. Squares denote high transmission sites that started SMC in 2014, triangles are moderate transmission sites that started SMC in 2019, and circles indicate low transmission sites that have not implemented SMC. **B** Therapeutic and chemoprevention drug use in Senegal. Therapeutic drug use in *blue/green*, chemoprevention drug use in *red/purple*. *SP* Sulfadoxine-Pyrimethamine, *AQ* amodiaquine, *ASAQ* Artesunate/Amodiaquine, *Coartem/AL* Coformulated Artemether Lumefantrine, *IPTp-SP* Intermittent preventative therapy in pregnant women using SP, *SMC* Seasonal malaria chemoprevention using SP + AQ.

*Plasmodium falciparum* samples from febrile patients collected between 2000 and 2020 were genotyped or sequenced. A total of 3,284 samples were collected from six sampling locations within Senegal: Pikine, Thiès, Kédougou, Diourbel, Kaolack, and Kolda (Fig. 1A). Kédougou and Kolda are high transmission regions in southeast Senegal. In 2021, reported annual incidence was 536.5 cases per 1000 in Kédougou and 214.5 cases per 1000 in Kolda [36]. Kaolack (10.9 cases per 1000) and Diourbel (19.4 cases per 1000) are intermediate transmission regions in central Senegal. Thiès (2.8 cases per 1000) and Pikine (4.9 cases per 1000) are low transmission sites in western Senegal.

The sample collection varied throughout time and space (Fig. S1, Supplemental Table 1). The 444 samples collected between 2000 and 2005 came exclusively from Pikine, the 800 samples collected between 2006 and 2014 came from Thiès, and the 340 samples collected between 2015 and 2017 came from Kédougou. Between 2017 and 2020, 1700 samples were collected from Thiès, Kaolack (starting in 2020), Diourbel, Kolda (starting in 2019), and Kédougou (continuously since 2015).

**Table 1:** Table of the molecular markers examined and their corresponding drug resistance phenotypes

Gene	SNP	Resistance
*Pfcrt*	K76T	Chloroquine, Amodiaquine
*Pfkelch13*	A578S, C580Y	Artemisinin
*Pfdhps*	A437G, K540E, A581G, A613T/S	Sulfadoxine
*Pfdhfr*	N51I, C59R, S108N	Pyrimethamine
*Pfmdr1*	N86Y, Y184F, D1246Y	Multiple, including Amodiaquine, Mefloquine, Chloroquine, Artemether-Lumefantrine

The sample collection spanned several important changes in official drug use policy (Fig. 1B). Changes in therapeutic anti-malarial treatment include the withdrawal of CQ, and successively, the introductions of SP, AQ, and ACT. Changes in chemoprevention strategies include the introduction of IPTp and SMC (Supplemental Tables 2 and 3). IPTp coverage at each of the examined study sites ranged between 33.3 and 73.2% from 2013 to 2022. SMC was introduced to Kolda and Kédougou in 2014, with SMC coverages ranging from 56.9 to 100%. Diourbel and Kaolack started implementing SMC in 2019. The reported SMC coverage for the health facility in Diourbel ranged from 44.9 to 53.9% and the health facility in Kaolack ranged from 57.3 to 82.2%.

Based on this drug policy history, several WHO-verified and validated SNP-based drug resistance markers in *Pfcrt*, *Pfdhfr*, *Pfdhps*, and *Pfmdr1* were examined (Table 1). For *Pfkelch13*, only *Pfkelch13* C580Y and A578S were examined. Because resistance to SP involves multiple mutations in *Pfdhfr* and *Pfdhps*, the frequency of *Pfdhfr* triple mutant IRN (N51**I**, C59**R**, and S108**N**) [37] and “quadruple” mutants (*Pfdhfr* triple mutant IRN + *Pfdhps* A437G) was examined. Likewise, the frequency of the *Pfmdr1* NFD (**N**86Y, Y184**F**, **D**1246Y) haplotype was also examined because this haplotype is associated with resistance to multiple drugs [19, 38]. Due in part to the long timescale of the molecular surveillance study, two different molecular genotyping approaches were used. High resolution melting assays were used for the mutations in *Pfcrt*, *Pfdhfr, Pfdhps* (**Methods**) while the mutations in *Pfkelch13* were examined using a more sensitive assay based on Sanger sequencing [25].

### Molecular surveillance detects rapid changes in *Pfcrt*, *Pfdhfr*, *Pfdhps*, and *Pfmdr1* mutations over time

**Fig. 2:**
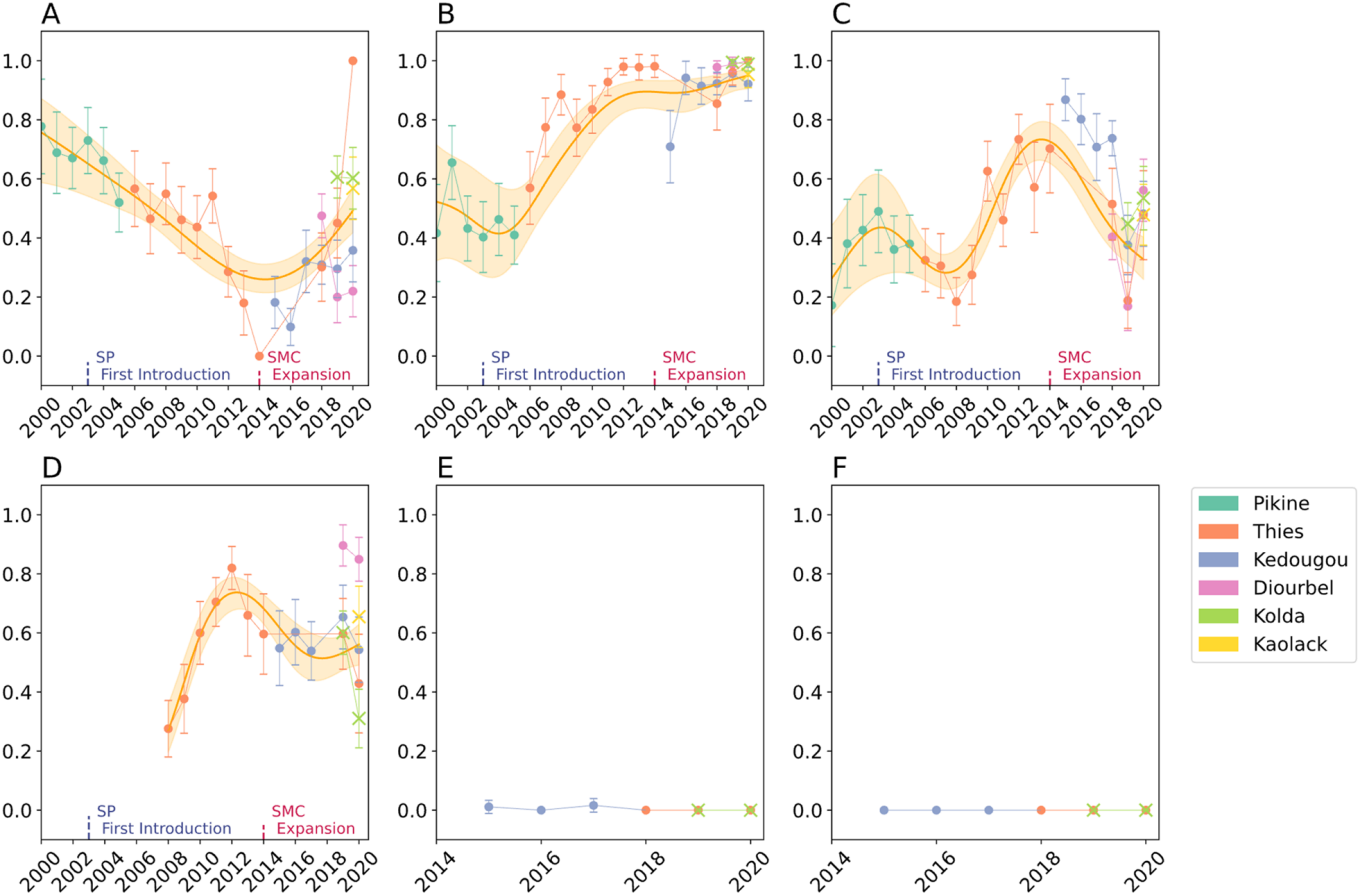
SNP-based Molecular Surveillance Results. Frequencies for **A** *Pfcrt* K76T, **B** *Pfdhfr* triple mutant (N51I, C59R, S108N), **C** *Pfdhps* A437G, **D** *Pfmdr1* N_86_F_184_D_1246_ haplotype (N86Y, Y184F, D1246Y), **E** *Pfkelch13* A578S, and **F**) *Pfkelch13* C580Y. The scatterplots show the observed frequencies and their 95% binomial confidence interval. Model predictions from a calibrated generalized additive model and the 95% confidence intervals are shown in orange. The model was calibrated with data from Pikine, Thiès, Diourbel, and Kédougou (denoted with circles). The data from Kolda and Kaolack (denoted with X) were not used for model calibration. Model predictions were not generated for the *Pfkelch13* mutations due to their complete or near-complete absence in the data.

Mutation frequencies in regions with more than three years of continuous sampling (Pikine, Thiès, and Kédougou, Fig. S2-S4) were first examined to identify any time-dependent trends. Pikine and Thiès are urban sites with low transmission that have not implemented SMC while Kédougou is a rural site with high transmission that has used SMC since 2014. The statistical significance of any temporal changes in mutation frequencies was assessed based on the p-values of the slope of a fitted, piecewise binomial generalized linear model. A binomial generalized additive model (GAM) was used to identify and highlight Senegal-wide trends in mutation frequency (**Methods**, Fig. 2). A GAM was used to summarize the data because it 1) provided a flexible framework to account for the stratified sampling of the molecular surveillance study, and 2) because the temporal changes in mutation frequency were not always monotonic and could either increase or decrease depending on the sampling period.

#### Increase in *Pfcrt* K76T mutation frequency after 2014

As expected, due to a fitness cost associated with *Pfcrt* K76T [39]*,* a statistically significant decline in *Pfcrt* K76T mutation frequency was observed between 2000 and 2014 (p-value < 0.001, Binomial generalized linear model) following the withdrawal of CQ in 2003 (Fig. 2A). Unexpectedly, this was followed by an increase in *Pfcrt* K76T frequency after 2014 (p-value < 0.001, Binomial generalized linear model). In 2000, the Senegal-wide GAM model estimated a Senegal-wide mutation frequency of 0.76 (95% CI 0.59—0.87), which fell to 0.26 (95% CI 0.21—0.31) in 2014. However, the frequency began rising after 2014, and the model estimated the frequency to be 0.49 (95% CI 0.41—0.57) in 2020. When evaluating the goodness-of-fit of the model, the adjusted *R*-squared for the model was 0.466 and the deviance explained was 54.2%. To account for differences in sample size, down-sampled estimates of mutation frequency were generated by re-estimating allele frequencies after down-sampling the data from each site-year to 29 samples, which was the smallest number of samples collected across all examined site-years involving Thies, Pikine, Kédougou, and Diourbel for the *Pfcrt K76T* mutation. Down-sampling had no effect on the trends in average mutation frequency over time (Fig. S5).

Closer examination of the changes in *Pfcrt* K76T in each of the individual populations revealed as a statistically significant increase in *Pfcrt* K76T in both Thiès and Kédougou (p-value < 0.001 for both, binomial generalized linear model) after 2014. When examining the sites not included in the Senegal-wide GAM (Kolda, and Kaolack), the frequency of *Pfcrt* K76T were consistent with those predicted by the GAM. However, the frequency of *Pfcrt* K76T in Diourbel declined (p-value < 0.001, Binomial generalized linear model) from 0.45 (95% CI 0.55—0.40) to 0.20 (95% CI 0.11—0.29) and 0.22 (95% CI 0.13—0.31) in 2018, 2019, and 2020, respectively.

#### Changes in *Pfdhfr* triple mutants following the withdrawal of CQ and introduction of SP

Based on the introduction of pyrimethamine in SP therapy in 2003 and IPTp chemoprevention in 2004, an increase in *Pfdhfr* IRN triple mutants over time was expected. The data revealed a sharp rise in the frequency of *Pfdhfr* triple mutants starting in 2003 (p-value < 0.001, Binomial generalized linear model), coinciding with the replacement of CQ with SP as the first-line antimalarial treatment in Senegal (Fig. 2B). The increase in the *Pfdhfr* triple mutant corresponded to a decrease in *Pfdhfr* triple sensitive alleles (N51N, C59C, S108S) (Fig. S6A) and only a few parasites with mixed haplotypes (different combinations of wild-type or mutant at the three amino acid sites) were observed. Across the entire dataset, only 0.06 (95% CI 0.057—0.075) of the samples had mixed haplotypes. In 2003, the GAM predicted that the frequency of triple mutants in Senegal was 0.42 (95% CI 0.27—0.59). By 2020, the predicted frequency was 0.95 (95% CI 0.90—0.97). The goodness of fit for the GAM model was evaluated using the adjusted R-squared and deviance, which were 0.881 and 89.4%, respectively.

#### Multiple increases and decreases in *Pfdhps* A437G frequency over time

Mutations in *Pfdhps* were expected to increase due to sulfadoxine exposure from either SP therapy or SP use in chemoprevention (IPTp or SMC). *Pfdhps* K540E, A581G, A613T/S were rare (< 5%) or undetected and only *Pfdhps* A437G was detected at a high frequency (Fig. 2C, Fig. S2-S4). Unlike with the *Pfdhfr* triple mutants, the *Pfdhps* A437G allele trajectory and “quadruple” mutant haplotype trajectory changed directions multiple times (Fig. S6B). Unusually, several inflection points were identified (at 2003, 2008, and 2014) where the trajectory of *Pfdhps* A437G prevalence changed direction.

Between 2000 and 2003, an increase in *Pfdhps* A437G was observed (p-value = 0.008, Binomial generalized linear model). The overall, Senegal-wide frequency of *Pfdhps* A437G as predicted by the GAM rose from 0.26 (95% CI 0.14—0.44) in 2000 to 0.47 (95% CI 0.19—0.77) in 2003. After 2003, *Pfdhps* A437G decreased until 2008, when its predicted frequency was 0.17 (95% CI 0.11—0.27). Between 2008 and 2014 (p-value = 0.005, Binomial generalized linear model), the frequency of *Pfdhps* A437G rose until 2014, where its GAM-predicted mutation frequency was 0.72 (95% CI 0.58—0.83). After 2014, the frequency of *Pfdhps* A437G declined (p-value = 0.022, Binomial generalized linear model), and the GAM-predicted mutation frequency in 2020 was 0.33 (95% CI 0.25—0.40). Overall, the adjusted *R*-squared and deviance explained by the model were 0.554 and 69.1%, respectively.

#### Molecular surveillance detects changes in *Pfmdr1* NFD haplotype over time

Expectations for the *Pfmdr1* NFD haplotype were less certain as mutations in *Pfmdr1* have been associated with resistance against multiple drugs. Between 2008 and 2012, a rapid increase in the *Pfmdr1* NFD (N86Y, Y184F, D1246Y) haplotype (Fig. 2D) was observed. The corresponding model-predicted frequencies were 0.27 (95% CI 0.20—0.37) in 2004 and 0.74 (95% CI 0.57—0.79) in 2012. After 2012, the predicted frequency of the *Pfmdr1* NFD haplotype declined to 0.56 (95% CI 0.48—0.63) in 2016, where it remained relatively stable until 2020 [0.56 (95% CI 0.49—0.63)]. The adjusted *R*-squared and deviance explained by the model were 0.817 and 87.2%, respectively.

#### Infrequent detection of *Pfkelch13* A578S in Senegal

The *Pfkelch13* A578S mutation was observed in Kédougou samples and identified in one of the 89 genotyped samples collected in 2015 and in two of the 123 genotyped samples collected in 2017, but in no other year or other site (Fig. 2E–F).

#### Genomic haplotype analyses reveal differences in selection acting on *Pfcrt*, *Pfdhps*, and *Pfdhfr*

To determine whether the allele frequency changes observed at *Pfcrt* K76T, *Pfdhps* A437G, and the *Pfdhfr* triple mutant IRN could be the result of selection, the genomic haplotypes surrounding these genes in a set of 231 samples collected from Thiès and Kédougou between 2006 and 2019 were examined from whole genome sequencing data (Fig. S7, Fig. S8). Strong, directional selection could result in selective sweeps that reduce the genomic haplotype diversity surrounding the mutation under selection. This reduction in genomic haplotype diversity can be quantified using a statistic known as the extended haplotype homozygosity (EHH) [11]; elevated EHH indicates a reduction in diversity (elevation of homozygosity) that could be the result of a selective sweep.

**Fig. 3:**
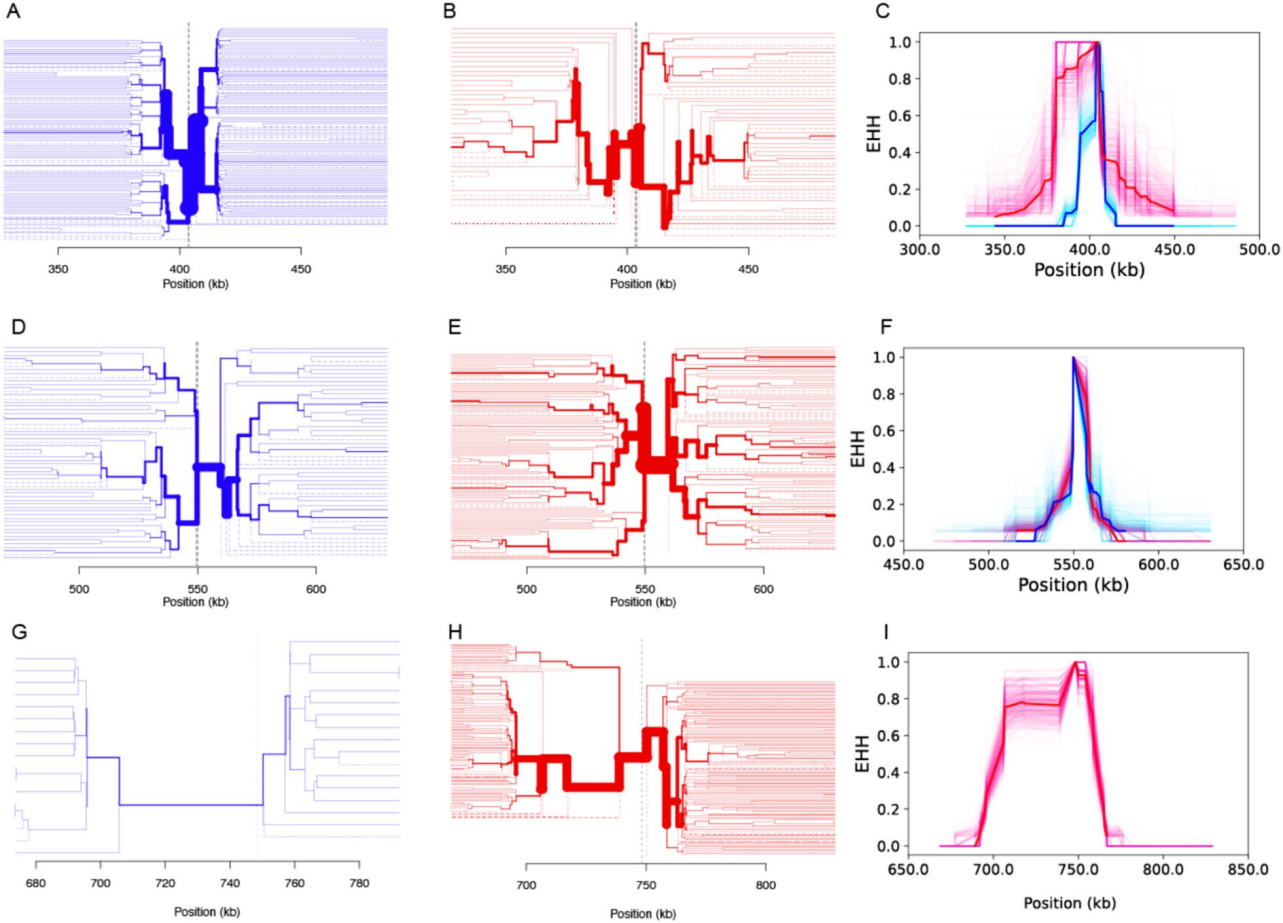
Evidence of selection at *Pfcrt, Pfdhps, *and* Pfdhfr*. Extended haplotype plots for the **A**–**C** *Pfcrt* K76T locus, **D**–**F** *Pfdhps* A437G locus, and **G-I** the *Pfdhfr* triple mutant. The first two columns are bifurcation plots that show the decay in haplotype structure around the focal locus. The first column (**A**, **D**, and **G**, *blue*) shows the haplotype structure surrounding the sensitive allele (*Pfcrt* K76*, Pfdhps* A437*, Pfdhfr* triple sensitive) while the second column (**B**, **E**, and **H**, *red*) shows the haplotype structure surrounding the resistance allele (*Pfcrt* K76T*, Pfdhps* A437G*,* and *Pfdhfr* triple resistant mutant. Starting from the center and extending outwards, each branch represents a bifurcation point where a new haplotype emerges. The thickness of the branch is proportional to the number of samples that contain that haplotype. The third column (**C**, **F**, and **I**) shows the EHH surrounding the sensitive (blue) and resistant (red) alleles. The solid red and blue lines are the EHH estimates obtained from using all available samples in the category. The lighter pink and blue traces are bootstrapped EHH estimates obtained by randomly downsampling fifty samples. For **G**–**I**, samples with mixed *Pfdhfr* genotypes were excluded.

Strong evidence of a selective sweep was identified around *Pfcrt* K76T. The haplotype structure surrounding *Pfcrt* K76T was far less diverse than that surrounding the ancestral *Pfcrt* K76 (Figs. 3A-3B). *Pfcrt* K76 haplotypes rapidly diversified within the first 20 kb. In contrast, *Pfcrt* K76T was surrounded by a dominant extended haplotype to its left (upstream, 5′) and two major extended haplotypes to its right (downstream, 3′). These dominant haplotypes extended out nearly 50 kb away from the *Pfcrt* K76T locus and included parasites collected before and after 2014*.* The EHH for *Pfcrt* K76T was elevated relative to *Pfcrt* K76 (Fig. 3C).

For *Pfdhps*, there was little evidence of a hard selective sweep (selection of a single or small number of haplotypes). Multiple long-range haplotypes extended outwards from either *Pfdhps* A437G or *Pfdhps* A437A for 30–50 kb and there was no significant elevation in EHH (Fig. 3D–F). The lack of EHH surrounding *Pfdhps *A437G, coupled with the strong changes in *Pfdhps* A437G mutation frequency over time, could indicate variable selective pressures that act as "soft sweeps" on multiple genomic haplotype backgrounds [12, 40]. To test this, two statistics were examined: H12, the expected haplotype homozygosity that assess the frequency of the two most common haplotypes, and H2/H1, the ratio of the second and the first most frequent haplotype (Fig. S9) [35]. Elevated H12 and a low H2/H1 ratio indicates a hard selective sweep while elevated H12 and a high H2/H1 ratio suggests a soft selective sweep (**Methods**). H12 around *Pfcrt* was high but H2/H1 was low, indicating a hard selective sweep (Fig. S9A). Both H12 and the H2/H1 ratio around *Pfdhps* were elevated (Fig. S9B), which is consistent with a soft selective sweep.

Strong extended haplotype structure was detected around the *Pfdhfr* triple mutant. Most *Pfdhfr* triple mutant parasites shared the same dominant haplotype (Fig. 3G–I). This haplotype extended out more than 50 kb to the left and 20 kb to the right of the *Pfdhfr* gene. The EHH of the *Pfdhfr* was likely due to a hard selective sweep, like that detected for *Pfcrt* K76T. However, this study lacked the sensitivity to accurately estimate the EHH surrounding the *Pfdhfr* triple sensitive genotype due to the rarity of *Pfdhfr* triple sensitive parasites. Most samples with usable *Pfdhfr* sequences were collected after 2010 (Fig. S6C), after the rapid increase in the *Pfdhfr* triple mutant identified by SNP-based molecular surveillance (Fig. 2C). *Pfdhfr* triple mutants comprised 0.83 (95% CI 0.77—0.89) of the whole genome sequenced samples; 0.07 (95% CI 0.03—0.11) were mixed mutants, and only 0.10 (95% CI 0.05—0.14) of the samples were *Pfdhfr* triple sensitive parasites.

#### Haplotype analyses reveal temporal changes in selection at *Pfcrt* K76T before and after 2014

**Fig. 4:**
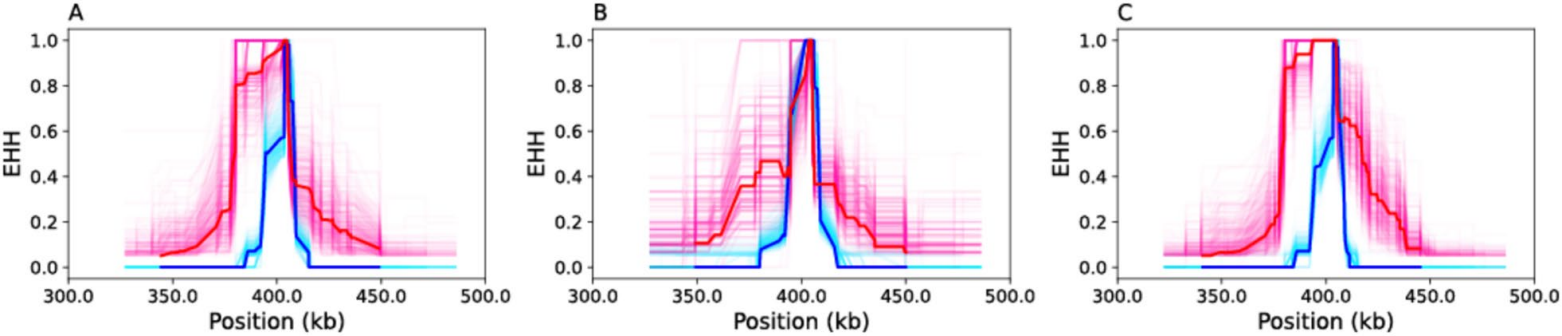
Temporal shifts in selection for *Pfcrt* K76T. Extended haplotype plots for the *Pfcrt* K76T locus using **A** all collected samples, **B** samples collected before or during 2014, and **C** samples collected after 2014. The solid red and blue lines are the EHH estimates obtained from using all available samples in the category. The lighter pink and blue traces are bootstrapped EHH estimates obtained by randomly downsampling fifty samples. Note that Fig. 4A is the same as Fig. 3C.

The rapid increase in *Pfcrt* K76T observed in the SNP-based molecular surveillance data after 2014 (Fig. 2A) suggested that the selective sweep surrounding *Pfcrt* K76T (Figs. 3C and 4A) was recent. To time the emergence of this selective sweep, two additional EHH analyses were performed on the whole genome sequencing data using data collected before and after 2014 (Fig. 4B, C). Prior to 2014, the EHH directly proximal to the *Pfcrt* K76T resistance mutation was similar to that observed surrounding the sensitive *Pfcrt* K76. However, elevated EHH was detected 10 kb upstream and downstream of the *Pfcrt* K76T locus, which is likely a legacy of the historical selective sweep that occurred prior to CQ withdrawal in 2003 (Fig. 4B) [41]. After 2014, the EHH surrounding *Pfcrt* K76T was elevated (Fig. 4A–C) and consistent with the expectations for a new selective pressure acting on the mutation occurring after 2014. These results further indicate that the elevated EHH surrounding *Pfcrt* K76T observed across all the samples (Figs. 3C and 4A) was driven by the post-2014 changes in EHH driven by this new selective sweep.

## Discussion

Accurate assessments of drug resistance in parasite populations are needed to ensure continued success of drug-based malaria control efforts. In this study, rapid changes in the mutation frequencies of drug resistance alleles were observed. These contrast with the stable allele trajectories seen at 24 neutral SNPs when surveying the genetic diversity of parasites in Thiès, Kédougou, and Richard Toll between 2012 and 2020 [42]. These changes are alarming and stress the need for follow-up experiments to confirm the drug resistance phenotypes of these parasites and new data collection strategies to attribute these changes in mutation frequencies to either difference in population structure (such as host age ranges) or therapeutic/prophylactic drug use.

In Senegal, *Pfdhfr* triple mutant approached near-fixation in 2013, which likely reflects pyrimethamine-mediated drug resistance from either anti-malarial SP therapy or from IPTp chemoprevention. A similar rise in *Pfdhfr* triple mutant frequency was previously reported by a study carried out in Thiès in 2003 and 2013 [43, 44]. The haplotype analyses show that the rapid fixation of *Pfdhfr* likely originated as a hard sweep acting on a single haplotype. The near-fixation of *Pfdhfr* triple mutant suggest that majority of parasites in Senegal have some degree of pyrimethamine resistance. Based on these results, SP chemoprevention efficacy should be closely monitored despite current WHO chemoprevention guidelines that recommend IPTp with SP in regions with high *Pfdhps* K540E frequencies [45].

These molecular surveillance results revealed two distinct trends in *Pfcrt* K76T allele frequency over time. Between 2003 and 2014, a decline in *Pfcrt* K76T was observed, which is consistent with the withdrawal of CQ the first-line anti-malarial therapy in Senegal due to widespread CQ resistance in Africa [41, 46, 47]. In the absence of CQ, *Pfcrt* K76T confers a fitness cost [48] and previous genomic surveillance studies have observed declines in *Pfcrt* K76T after the withdrawal CQ [39, 49, 50] and similar reductions in EHH (Fig. 4B) surrounding the *Pfcrt* K76T mutation as parasites begin outcrossing more frequently with CQ sensitive parasites [51, 52].

After 2014, this study showed an increase in *Pfcrt* K76T mutation frequency that was accompanied by evidence of a new selective sweep starting in 2014. *Pfcrt* K76T is most associated with CQ resistance, but it is unlikely that CQ use is responsible for the observed rise in *Pfcrt* K76T. While unofficial CQ use during the COVID-19 pandemic has been reported [53], the rise of *Pfcrt* K76T predates the COVID-19 pandemic. Instead, the increase in *Pfcrt* K76T likely reflects increasing AQ resistance [54–56]. These results are troubling, as AQ is used as a partner drug for ACT (as artesunate-amodiaquine [ASAQ]) and for chemoprevention in SMC. Mathematical models predict a decline in ASAQ treatment efficacy when *Pfcrt* K76T frequencies are high [57] or when partner drug resistance is high [58]. Fortunately, recent TES studies in Senegal show that ASAQ remains effective [59, 60], but these molecular surveillance results suggest that the efficacy of AQ as a partner drug for ACT or for chemoprevention may be at risk of decline in the future. Given these risks, therapeutic ASAQ treatment in regions targeted for SMC should be avoided.

A variety of mutations and copy number variations in *Pfmdr1* gene have been shown to modulate resistance against multiple drugs as well as CQ and AQ [19]. The *Pfmdr1* haplotype NFD was the most dominant genotype in this Senegal parasite population with an overall increase in frequency from 0.2 in 2004 to 0.56 in 2020. This haplotype has previously been reported to be associated with artemether-lumefantrine (AL) treatment failure, associated with in vitro reduced AL sensitivity [61–63], and used as a surrogate marker for AQ and lumefantrine resistance [17]. This result suggests that the use of AL in this region might be selecting for these specific *Pfmdr1* alleles and the changes in haplotype frequency may reflect the combined impact of multiple drugs. The factors driving the changes in *Pfmdr1* and their implications for future therapeutic and chemoprevention strategies is a subject for future study.

Likewise, the rapid frequency changes at *Pfdhps* A437G was unexpected and different from the accumulation of “quadruple” mutant parasites (parasites that are *Pfdhfr* triple mutant and *Pfdhps* A437G) observed in African countries where SP is administered [64]. These results are more difficult to interpret but could reflect fluctuating drug-mediated pressure over time. While *Pfdhps* A437G is associated with sulfadoxine resistance, it is possible that the various additional introductions of SP-AQ, ASAQ, and Coartem/AL [65] during this time period have altered the fitness benefits and costs associated with *Pfdhps* A437G over time. The data also suggested a possible rebound in *Pfdhps* A437G starting in 2018 that could not be captured by the current Senegal-wide GAM model because there is insufficient data after 2018 to properly fit the model. Refitting the Senegal-wide model to additional data from after 2020 will be needed to determine the significance of this rebound and determine whether it continues into the future. More detailed assessments of drug use and phenotypic studies are needed to determine whether these changes are related to an AQ-induced adaptation cost or other adaptive change.

Previous studies across the African continent have identified changes in *Pfcrt,* mutations in *Pfdhps* and *Pfdhfr* that are associated with the use of SMC [66–68]. High frequencies of *Pfdhps* A437G, as part of the *Pfdhps* VAGKS (431 V-436A-437G-540 K-581G-613S) haplotype, and *Pfcrt* K76T, as part *Pfcrt* CVIET (72C-73 V-74I-75G-76 T) haplotype have been reported in countries using SMC. This study showed that the near fixation of *Pfdhfr* triple mutant in Senegal predated SMC in Senegal and showed that the rise in *Pfcrt* K76T coincided with a new selective sweep in 2014, the same year that SMC was first implemented in Senegal. Detailed follow-up analysis of the malaria clinical epidemiology and drug usage patterns are needed to confirm to what extent these changes in drug resistance markers can be attributed to drug usage in Senegal.

A major limitation of this study was that sample collection was uneven across space and time. Despite this, common changes in mutation frequency across multiple sites in Senegal were observed. For example, changes in *Pfdhps* A437G and *Pfcrt* K76T were observed in both Kédougou where SP-AQ (SMC) is applied and AL is used therapeutically, and in the non-SMC region of Thiès where ASAQ is used as treatment. Reports from the 2014 Senegal census show significant levels of human movement from the higher transmission, southeastern corner of the country (Kédougou) to the lower transmission western sections of the country (Thiès) [69], which could explain why similar changes in drug resistance mutation frequency were observed in multiple collection sites. Previous parasite population genetic analyses of neutral or highly polymorphic markers and patterns of genetic relatedness in Senegal have consistently suggested a well-mixed parasite population [28, 42, 70], unlike the fragmented parasite populations observed in the Greater Mekong region of Southeast Asia [71].

The consistency of the changes in allele frequency across sampling sites motivated the use of a single, Senegal-wide GAM model to identify Senegal-wide trend across all the study sites. Assuming a Senegal-wide trend was based on the drug resistance marker data from Pikine, Thiès, and Kédougou, the geographical proximity of (~ 30 miles) Pikine and Thiès, and the consistency of post-2014 trends across all examined study sites, including Kolda and Kaolack. Overall, the model-estimated allele frequency trajectories mirrored those observed in the actual data and can be useful for summarizing molecular surveillance results in studies with significant stratification in sampling with respect to space and time. However, this approach should only be used when there is a high degree of connectivity between sampling locations because the model assumes that each location represents a sampling from a greater, country-wide trend. This framework could also be used to identify sites whose molecular surveillance results appear to be deviating from the general, country-wide trend. For example, Diourbel was the only study site where the frequency of *Pfcrt* K76T was observed to be declining since 2014 and suggests that there is something unique about Diourbel relative to the rest of Senegal. Previous population genomic analyses in Diourbel have revealed an unusually high clonal parasite population genetic structure relative to the rest of the Senegal population [28].

When examining mutations associated with ART resistance, this study was limited to only those mutations in the *Pfkelch13* propeller domain. With the exception of *Pfkelch13* A578S, no other mutations in the *Pfkelch13* propeller domain were observed [20–22]. A previous study using targeted deep amplicon sequencing performed on the *Pfkelch13* gene in Senegalese parasites did not identify any additional mutations associated with ART resistance [72]. Other *Pfkelch13* mutations (C469Y and A675V) that have been associated with ART resistance in Uganda [23, 73, 74] were not observed. As of this study, TES studies in Senegal show that ACTs remain highly effective [59, 60], but the recent emergence of African ART resistance mutations highlight the need to continuously assess ART resistance risk in Senegal.

Molecular surveillance opens up a unique opportunity to assess changing drug risk before it causes a decline in clinical efficacy. Given that loss of ACT drug efficacy has historically been preceded by both ART resistance and partner drug resistance, molecular surveillance offers an opportunity to assess emerging drug resistance before efficacy has been compromised. In Senegal, the sudden increase in *Pfcrt* K76T after 2014 highlights the potential risk of emerging AQ resistance that could threaten the future efficacy of ASAQ-based therapy and SMC-based chemoprevention.

### Supplementary Information

Supplementary Material 1: Fig. S1 Sample sizes for SNP-based molecular surveillance. Sample size per year per region for the SNP-based molecular surveillance. Fig. S2 SNP-based molecular surveillance in Pikine for A) *Pfcrt*, B) *Pfdhfr*, C) *Pfdhps* and D) *Pfmdr1*. The *Pfdhfr* I174L and *Pfkelch13* SNPs were not examined in Pikine. Error bars indicate two binomial standard deviations from the mean. X’s denote years where samples were collected but the mutation was not observed. Gaps in the data were because samples were not collected for that year. Fig. S3 SNP-based molecular surveillance in Thiès for A) *Pfcrt*, B) *Pfdhfr*, C) *Pfdhps*, D) *Pfmdr1*, and E) *Pfkelch13*. Error bars indicate two binomial standard deviations from the mean. X’s denote years where samples were collected but the mutation was not observed. Gaps in the data were because samples were not collected for that year. Fig. S4 SNP-based molecular surveillance in Kédougou for A) *Pfcrt*, B) *Pfdhfr*, C) *Pfdhps*, D) *Pfmdr1*, and E) *Pfkelch13*. Error bars indicate two binomial standard deviations from the mean. X’s denote years where samples were collected but the mutation was not observed. Gaps in the data were because samples were either not collected or not genotyped for that year. Fig. S5 Down-sampled estimates of *Pfcrt* K76T for A) Thies, B) Pikine, C) Kédougou, and E) Diourbel. In blue are the estimated allele frequencies and 95% confidence intervals obtained from the raw data. In grey are average allele frequencies and 95% confidence intervals after down-sampling the data from each site-year to 29 samples, which was the smallest number of samples collected across all examined site-years involving Thies, Pikine, Kédougou, and Diourbel. Fig. S6 A) Frequency of *Pfdhfr* triple sensitive (N51, C59, S108) parasites. B) Frequency of “quadruple” (*Pfdhfr* triple mutant + *Pfdhps* A437G) parasites. The scatterplots show the observed frequencies and their 95% binomial confidence interval. Model predictions from a calibrated generalized additive model and the 95% confidence intervals are shown in orange. The model was calibrated with data from Pikine, Thiès, Diourbel, and Kédougou (denoted with circles). The data from Kolda and Kaolack (denoted with X) were not used for model calibration. Fig. S7 Sampling distribution for our whole genome sequence collection (A). Grey indicates that the sample came from Thiès. Green indicates the sample came from Kédougou. Sampling distributions for B) the *Pfcrt* genomic region, C) the *Pfdhfr* genomic region, and D) the *Pfdhps* genomic regions. For B and D, blue denotes samples with the sensitive allele and red indicates those with the resistance allele. For C, red denotes samples that are *Pfdhfr* triple mutant, blue indicates those that are *Pfdhfr* triple sensitive, and orange indicates those with a mix of resistant and sensitive alleles at the three examined *Pfdhfr* loci. Fig. S8 SNPs used to define genomic haplotypes. Genomic haplotypes surrounding the wild-type mutations: A) *Pfcrt* K76, B) *Pfdhfr* C59, C) *Pfdhps* A437 and the drug resistance mutations: D) *Pfcrt* K76T, E) *Pfdhfr* C59R, F) *Pfdhps* A437G. 171 samples and 173 SNPs were examined for *Pfcrt*, 170 samples and 85 SNPs for *Pfdhfr*, 182 samples and 83 SNPs for *Pfdhps*. Each row represents a sample. The left most column indicates whether the sample was collected before or during 2014 (green) or after 2014 (yellow). Alleles corresponding to the 3D7 reference are indicated by light blue and alleles corresponding to the alternative allele are indicated by dark blue. White corresponds to missing data. The orange boxes highlight the boundaries of the *Pfcrt* (A/D), *Pfdhfr* (B/E), and *Pfdhps* (C/F) genes. Fig. S9 Evidence of Hard and Soft Sweeps H12 (blue, left y-axis) and H2/H1 (orange, right y-axis) statistics for A) chromosome 7 and B) chromosome 8. The dotted green lines show the location of *Pfcrt* or *Pfdhps*. Table 1. Sample sizes for SNP-based molecular surveillance. Table 2. Regional and health facility IPTp coverage where samples were collected: Pikine (Deggo), Thiès (SLAP), Diourbel (Sessene), Kaolack (P. Assainies), Kolda (Bagadadji), and Kédougou (Bandafassi). Table 3. Regional SMC and health facility coverage where samples were collected: Pikine (Deggo), Thiès (SLAP), Diourbel (Sessene), Kaolack (P. Assainies), Kolda (Bagadadji), and Kédougou (Bandafassi).

## Data Availability

All relevant data and code will be made available on publication. SNP data and relevant analyses code will be made available in a GitHub repository. Sequence data are being uploaded to the NCBI Sequence Read Archive under BioProject PRJNA972644. All genetic and genomic data from this study are provided by the Senegal Ministry of Health and Social Action and made publicly available, along with the population data used in this study. These population data include the date of sample collection, the name of the health facility where the sample was collected, and the latitude and longitude of the health facility. No other sample information (i.e., related to the individual who provided the sample) was utilized as per the ethical approval for the study.

## Abbreviations

ACT: Artemisinin-based combination therapy
AL: Artemether-lumefantrine
AQ: Amodiaquine
ART: Artemisinin
ASAQ: Artesunate-amodiaquine
CQ: Chloroquine
EHH: Extended haplotype homozygosity
GAM: Generalized Additive Model
IPTp: Intermittent preventative treatment in pregnant women
IPTi: Intermittent preventative treatment in infants
MDA: Mass drug administration
SMC: Seasonal malaria chemoprevention
SNP: Single nucleotide polymorphism
SP: Sulfadoxine-pyrimethamine
TES: Therapeutic efficacy studies
WHO: World Health Organization

## Code availability

All relevant code for analysis will be made available on Github at (https://github.com/weswong/senegal_drugR_2000-2020) and archived in Zenodo upon publication.
