# Supplementary material for: Two decades of molecular surveillance in Senegal reveal rapid changes in known drug resistance mutations over time
Source: Malar J. 2024 Jul 9;23:205. doi: 10.1186/s12936-024-05024-8 (PMC11234717; doi:10.1186/s12936-024-05024-8)
Supplement: Supplementary file 1 — Supplementary Material 1: Fig. S1 Sample sizes for SNP-based molecular surveillance. Sample size per year per region for the SNP-based molecular surveillance. Fig. S2 SNP-based molecular surveillance in Pikine for A) Pfcrt, B) Pfdhfr, C) Pfdhps and D) Pfmdr1. The Pfdhfr I174L and Pfkelch13 SNPs were not examined in Pikine. Error bars indicate two binomial standard deviations from the mean. X’s denote years where samples were collected but the mutation was not observed. Gaps in the data were because samples were not collected for that year. Fig. S3 SNP-based molecular surveillance in Thiès for A) Pfcrt, B) Pfdhfr, C) Pfdhps, D) Pfmdr1, and E) Pfkelch13. Error bars indicate two binomial standard deviations from the mean. X’s denote years where samples were collected but the mutation was not observed. Gaps in the data were because samples were not collected for that year. Fig. S4 SNP-based molecular surveillance in Kédougou for A) Pfcrt, B) Pfdhfr, C) Pfdhps, D) Pfmdr1, and E) Pfkelch13. Error bars indicate two binomial standard deviations from the mean. X’s denote years where samples were collected but the mutation was not observed. Gaps in the data were because samples were either not collected or not genotyped for that year. Fig. S5 Down-sampled estimates of Pfcrt K76T for A) Thies, B) Pikine, C) Kédougou, and E) Diourbel. In blue are the estimated allele frequencies and 95% confidence intervals obtained from the raw data. In grey are average allele frequencies and 95% confidence intervals after down-sampling the data from each site-year to 29 samples, which was the smallest number of samples collected across all examined site-years involving Thies, Pikine, Kédougou, and Diourbel. Fig. S6 A) Frequency of Pfdhfr triple sensitive (N51, C59, S108) parasites. B) Frequency of “quadruple” (Pfdhfr triple mutant + Pfdhps A437G) parasites. The scatterplots show the observed frequencies and their 95% binomial confidence interval. Model predictions from a calibrated generalize [file 12936_2024_5024_MOESM1_ESM.docx]

**Supplemental Figures**


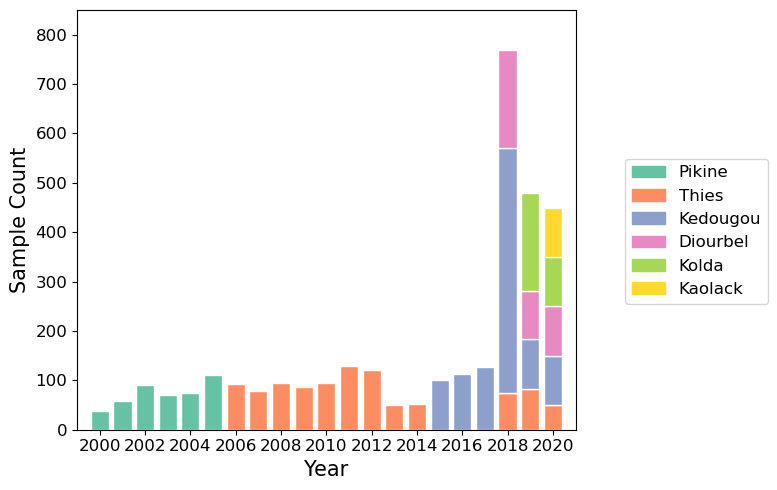


**Fig. S1 Sample sizes for SNP-based molecular surveillance.** Sample size per year per region for the SNP-based molecular surveillance.


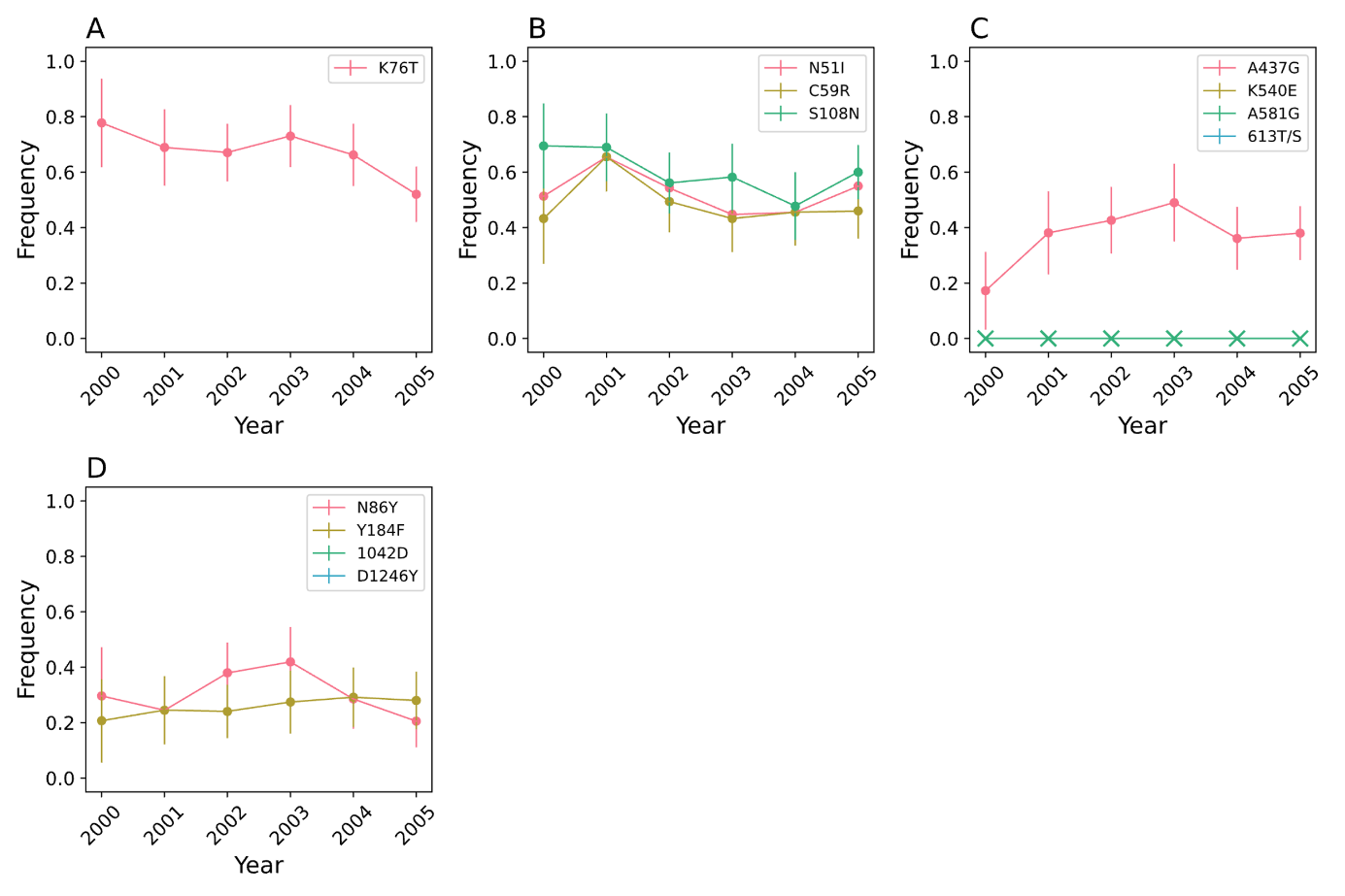


**Fig. S2 SNP-based molecular surveillance in Pikine** for **A**) *Pfcrt*, **B**) *Pfdhfr*, **C**) *Pfdhps* and **D**) *Pfmdr1*. The *Pfdhfr I174L* and *Pfkelch13* SNPs were not examined in Pikine. Error bars indicate two binomial standard deviations from the mean. X’s denote years where samples were collected but the mutation was not observed. Gaps in the data were because samples were not collected for that year.

**
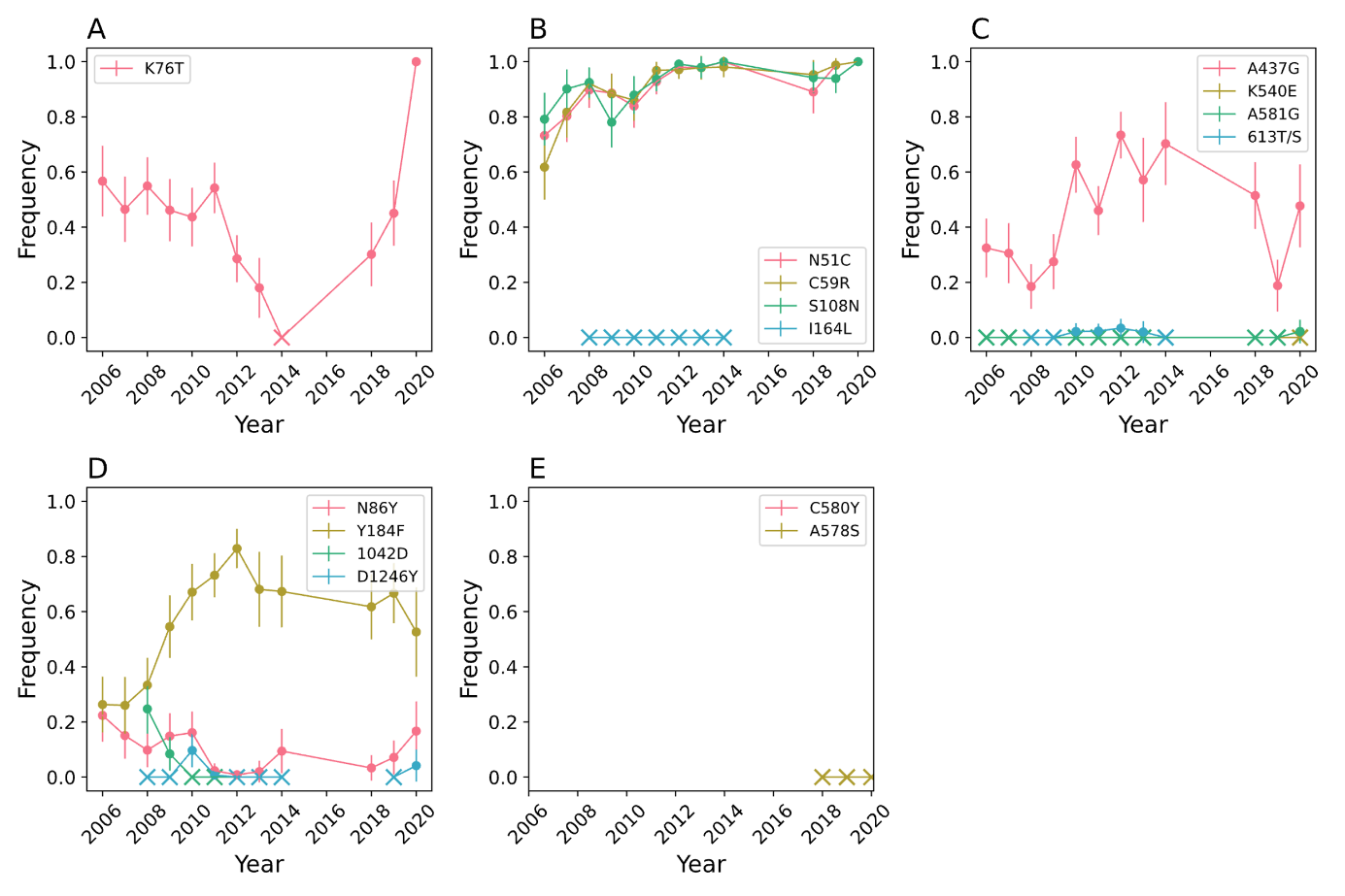
**

**Fig. S3 SNP-based molecular surveillance in Thiès** for **A**) *Pfcrt*, **B**) *Pfdhfr*, **C**) *Pfdhps*, **D**) *Pfmdr1*, and **E**) *Pfkelch13*. Error bars indicate two binomial standard deviations from the mean. X’s denote years where samples were collected but the mutation was not observed. Gaps in the data were because samples were not collected for that year.


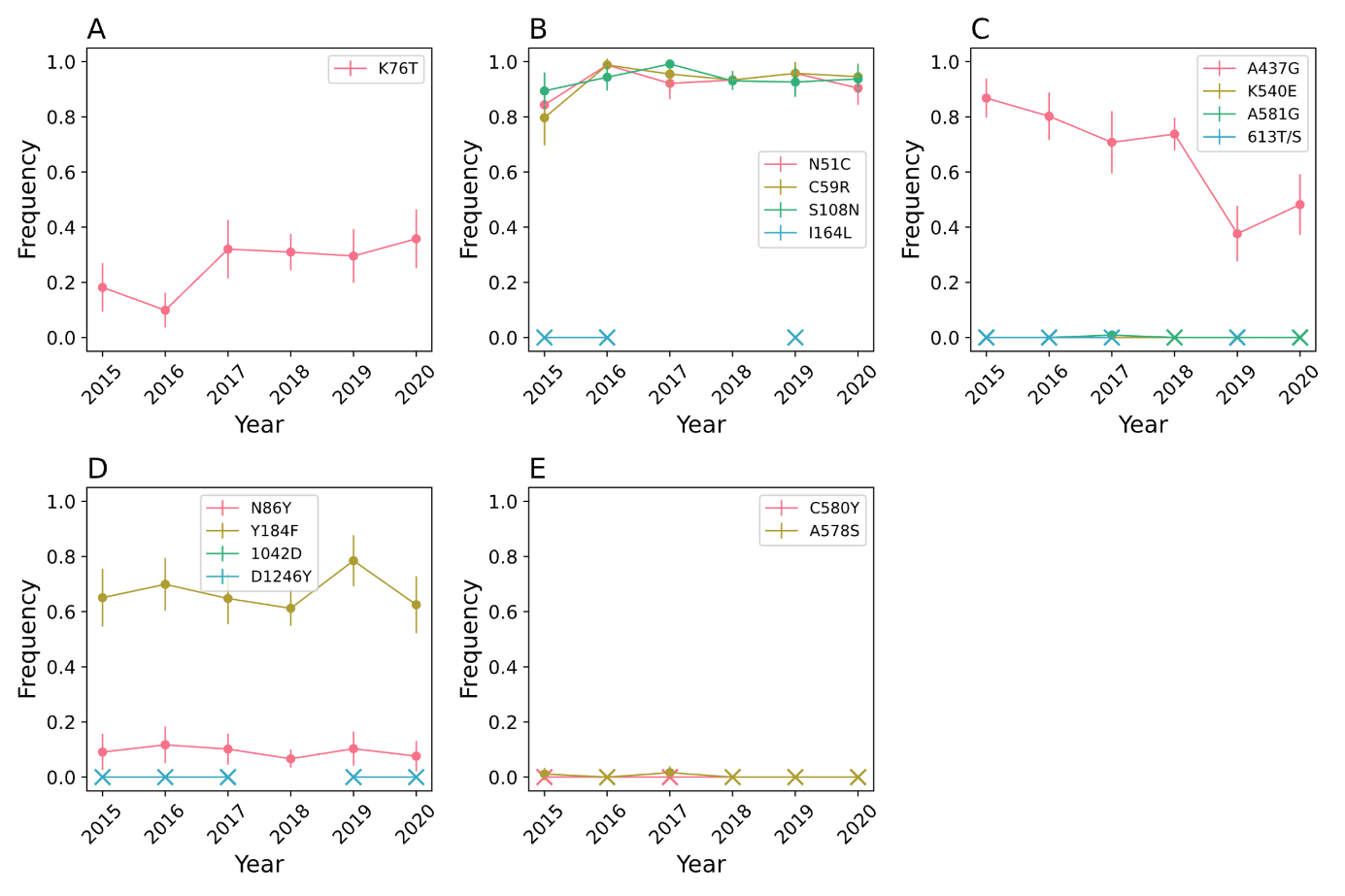


**Fig. S4 SNP-based molecular surveillance in Kédougou** for **A**) *Pfcrt*, **B**) *Pfdhfr*, **C**) *Pfdhps*, **D**) *Pfmdr1*, and **E**) *Pfkelch13*. Error bars indicate two binomial standard deviations from the mean. X’s denote years where samples were collected but the mutation was not observed. Gaps in the data were because samples were either not collected or not genotyped for that year.


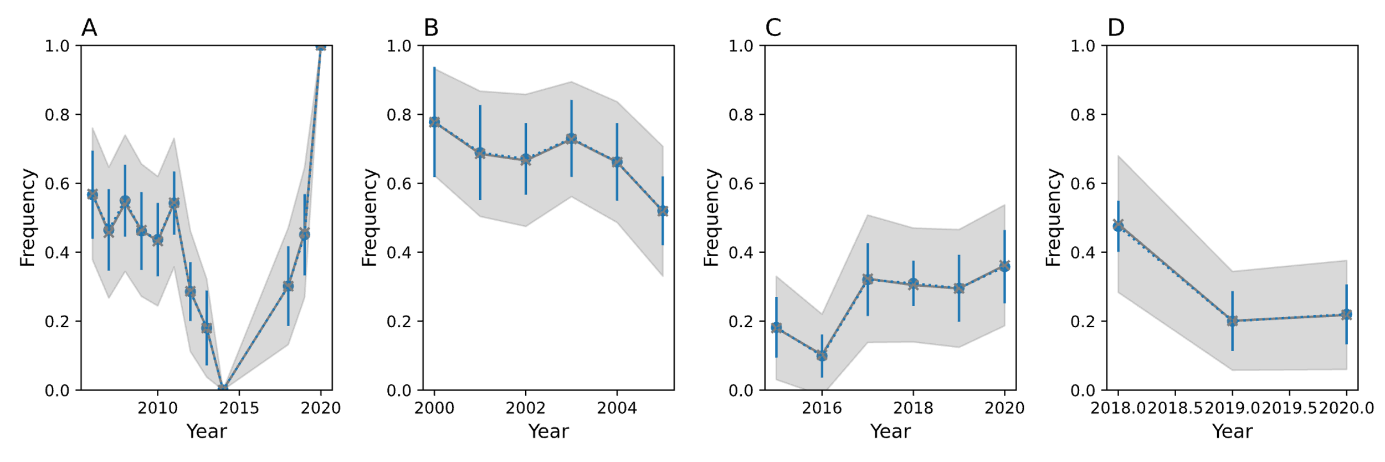


**Fig. S5** **Down-sampled estimates of *Pfcrt K76T*** for **A**) Thies, **B**) Pikine, **C**) Kédougou, and **E**) Diourbel. In blue are the estimated allele frequencies and 95% confidence intervals obtained from the raw data. In grey are average allele frequencies and 95% confidence intervals after down-sampling the data from each site-year to 29 samples, which was the smallest number of samples collected across all examined site-years involving Thies, Pikine, Kédougou, and Diourbel.


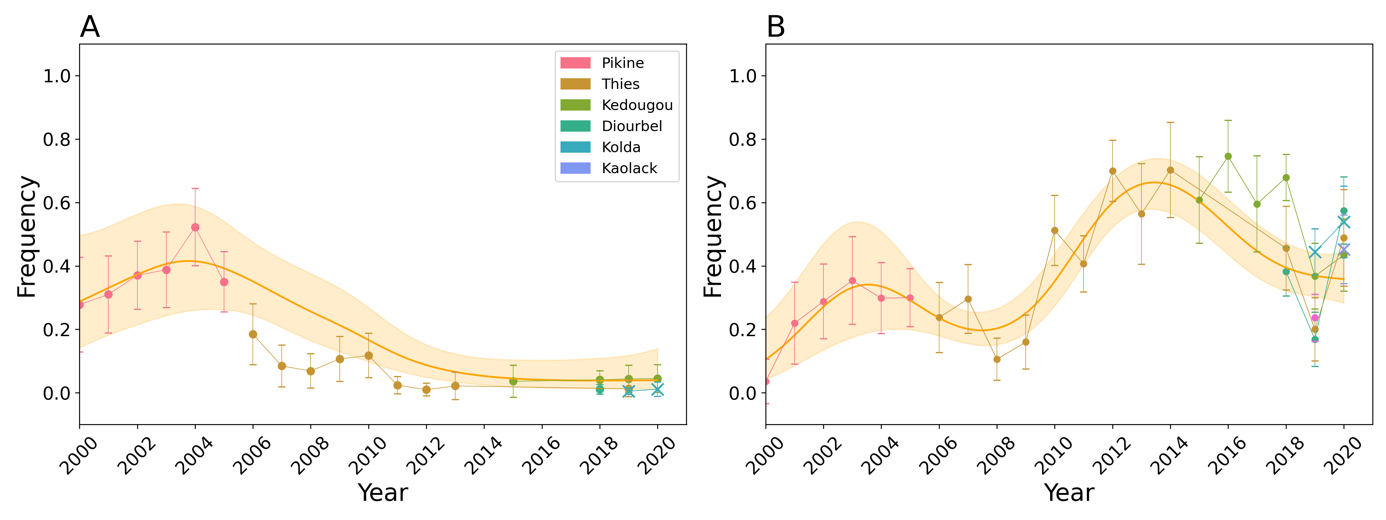


**Fig. S6 A**) Frequency of *Pfdhfr* triple sensitive (N51, C59, S108) parasites. **B**) Frequency of “quadruple” (*Pfdhfr* triple mutant + *Pfdhps* A437G) parasites. The scatterplots show the observed frequencies and their 95% binomial confidence interval. Model predictions from a calibrated generalized additive model and the 95% confidence intervals are shown in orange. The model was calibrated with data from Pikine, Thiès, Diourbel, and Kédougou (denoted with circles). The data from Kolda and Kaolack (denoted with X) were not used for model calibration.


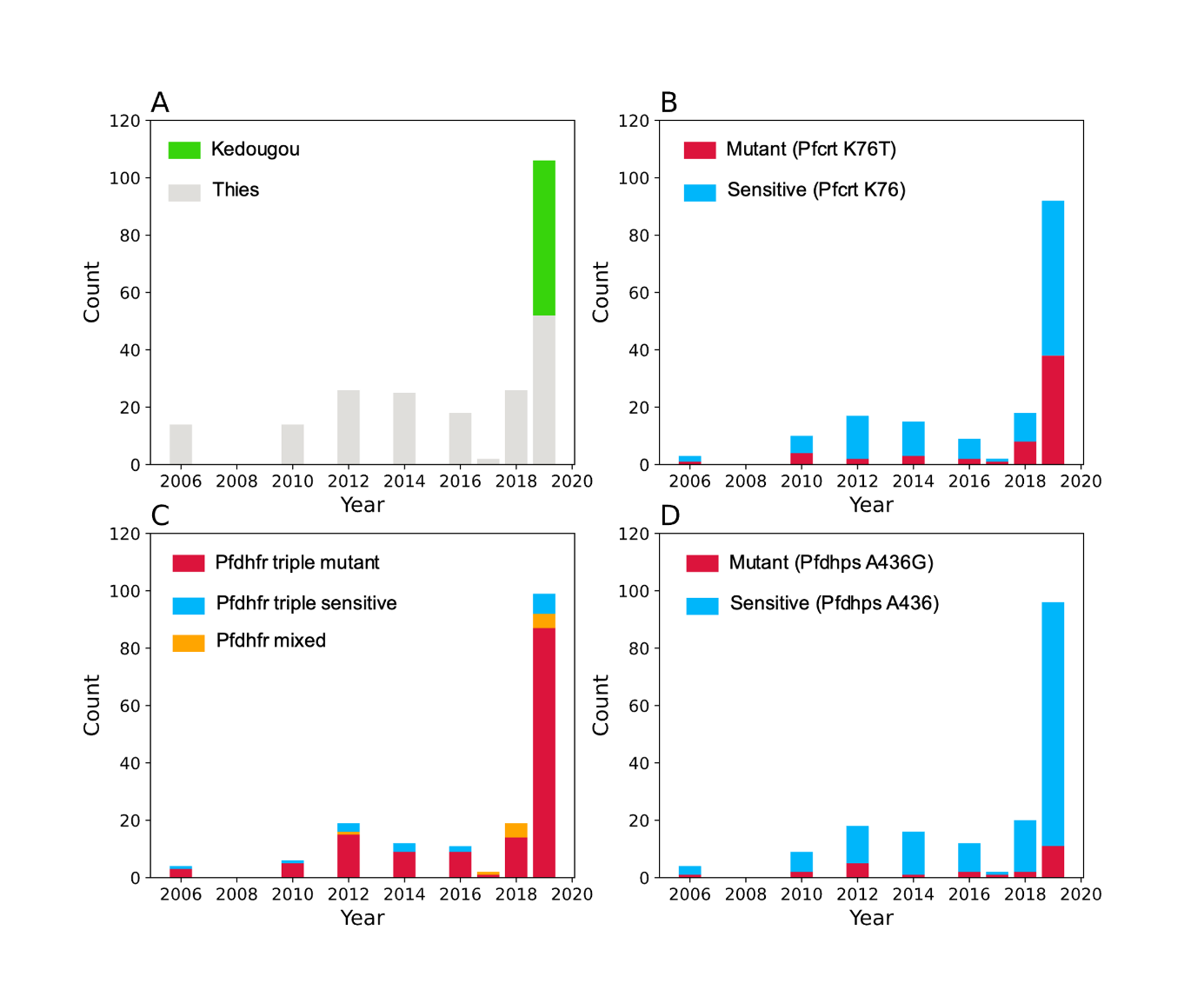


**Fig. S7 Sampling distribution for our whole genome sequence collection** (**A**). Grey indicates that the sample came from Thiès. Green indicates the sample came from Kédougou. Sampling distributions for **B**) the *Pfcrt* genomic region, **C**) the *Pfdhfr* genomic region, and **D**) the *Pfdhps* genomic regions. For **B** and **D**, blue denotes samples with the sensitive allele and red indicates those with the resistance allele. For **C**, red denotes samples that are *Pfdhfr* triple mutant, blue indicates those that are *Pfdhfr* triple sensitive, and orange indicates those with a mix of resistant and sensitive alleles at the three examined *Pfdhfr* loci.


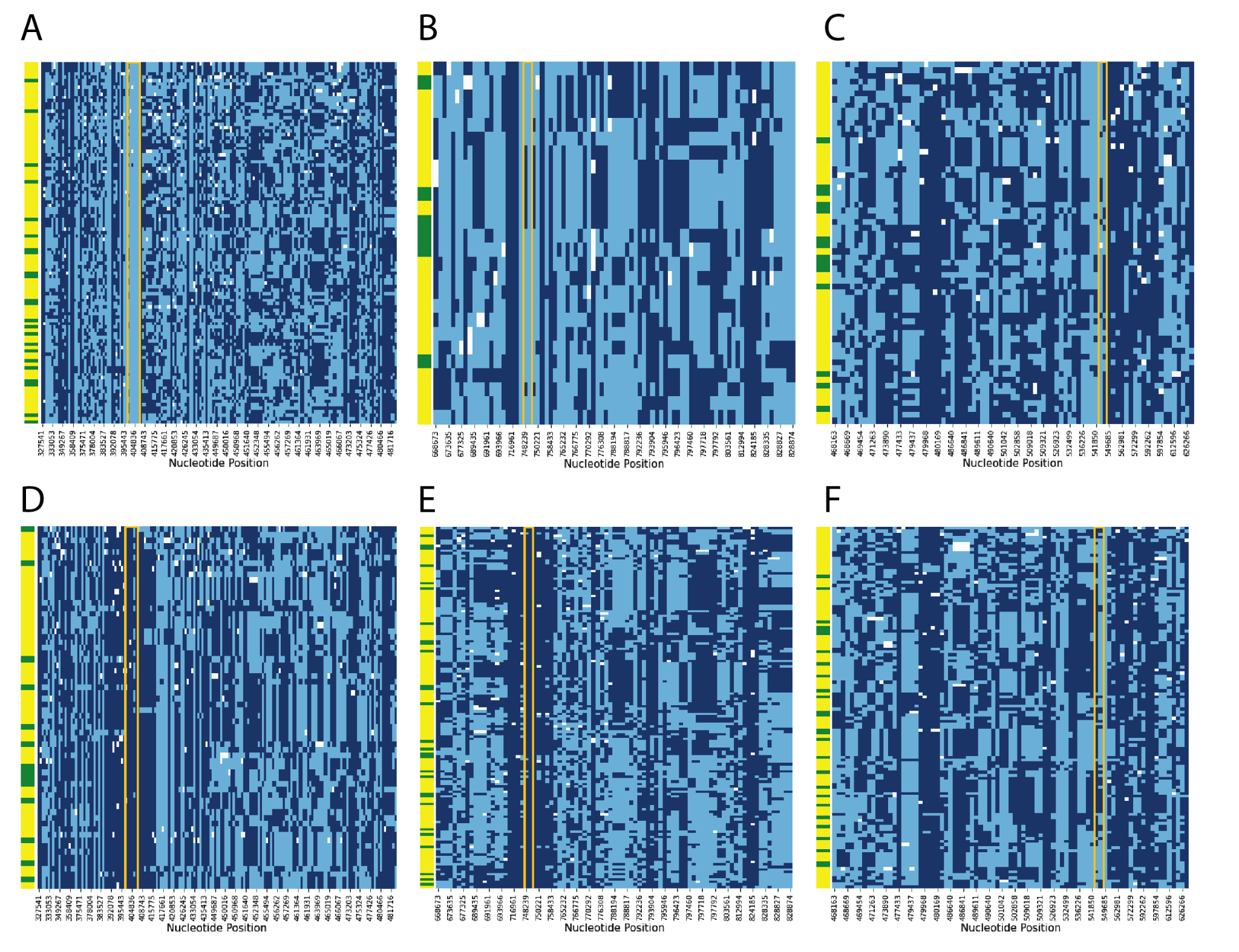


**Fig. S8 SNPs used to define genomic haplotypes.** Genomic haplotypes surrounding the wild-type mutations: **A**) *Pfcrt* K76, **B**) *Pfdhfr* C59, **C**) *Pfdhps* A437 and the drug resistance mutations: **D**) *Pfcrt* K76T, **E**) *Pfdhfr* C59R, **F**) *Pfdhps* A437G. 171 samples and 173 SNPs were examined for *Pfcrt*, 170 samples and 85 SNPs for *Pfdhfr*, 182 samples and 83 SNPs for *Pfdhps*. Each row represents a sample. The left most column indicates whether the sample was collected before 2014 (*green*) or after 2014 (*yellow*). Alleles corresponding to the 3D7 reference are indicated by *light blue* and alleles corresponding to the alternative allele are indicated by *dark blue*. White corresponds to missing data. The orange boxes highlight the boundaries of the *Pfcrt* (**A/D**), *Pfdhfr* (**B/E**), and *Pfdhps* (**C/F**) genes,


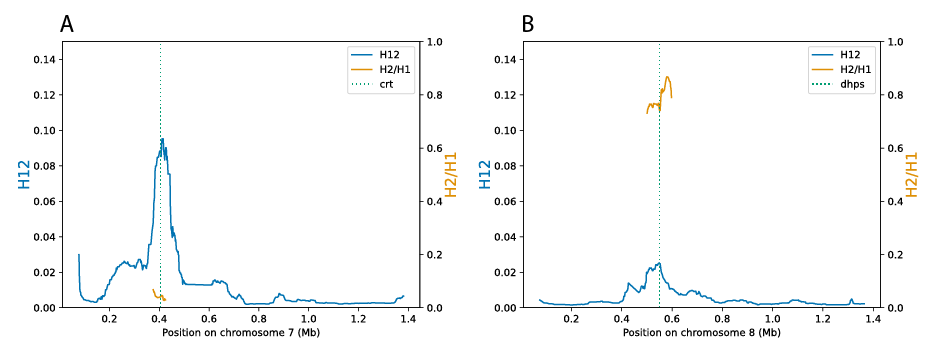


**Fig. S9 Evidence of Hard and Soft Sweeps** H12 (*blue*, left y-axis) and H2/H1 (*orange*, right y-axis) statistics for **A**) chromosome 7 and **B**) chromosome 8. The dotted green lines show the location of *Pfcrt* or *Pfdhps*.

**Supplemental Tables**

**Supplemental Table 1** Sample sizes for SNP-based molecular surveillance.

| **Year** | **Pikine** | **Thies** | **Kaolack** | **Diourbel** | **Kolda** | **Kedougou** |
| --- | --- | --- | --- | --- | --- | --- |
| 2000 | 38 |  |  |  |  |  |
| 2001 | 59 |  |  |  |  |  |
| 2002 | 91 |  |  |  |  |  |
| 2003 | 71 |  |  |  |  |  |
| 2004 | 74 |  |  |  |  |  |
| 2005 | 111 |  |  |  |  |  |
| 2006 |  | 92 |  |  |  |  |
| 2007 |  | 79 |  |  |  |  |
| 2008 |  | 94 |  |  |  |  |
| 2009 |  | 87 |  |  |  |  |
| 2010 |  | 95 |  |  |  |  |
| 2011 |  | 130 |  |  |  |  |
| 2012 |  | 120 |  |  |  |  |
| 2013 |  | 50 |  |  |  |  |
| 2014 |  | 53 |  |  |  |  |
| 2015 |  |  |  |  |  | 100 |
| 2016 |  |  |  |  |  | 112 |
| 2017 |  |  |  |  |  | 128 |
| 2018 |  | 74 |  | 200 |  | 496 |
| 2019 |  | 83 |  | 97 | 200 | 100 |
| 2020 |  | 50 | 100 | 100 | 100 | 100 |

**Supplemental Table 2** Regional and health facility IPTp coverage where samples were collected: Pikine (Deggo), Thiès (SLAP), Diourbel (Sessene), Kaolack (P. Assainies), Kolda (Bagadadji), and Kédougou (Bandafassi).

**Supplemental Table 3** Regional SMC and health facility coverage where samples were collected: Pikine (Deggo), Thiès (SLAP), Diourbel (Sessene), Kaolack (P. Assainies), Kolda (Bagadadji), and Kédougou (Bandafassi).
